# Supplementary material for: Melanoma in Peru: 1000 patients and 10 years of experience
Source: PLoS One. 2025 Oct 8;20(10):e0323636. doi: 10.1371/journal.pone.0323636 (PMC12507319; doi:10.1371/journal.pone.0323636)
Supplement: Attachment — Submitted filename: Melanoma 1000 pts Response to reviewers.docx [file pone.0323636.s004.docx]

**Response to reviewers**

**Reviewer’s comment:**

Excellent work and I congratulate the authors for their hardwork.

**Authors’ response:**

Thank you very much for the kind words of the reviewer, we really appreciate it.

**Reviewer’s comment:**

Please clarify the following..

**1.**How follow up was done for 10 long years?

**Authors’ response:**

Thank you for this comment, our follow up was done according to NCCN guidelines. After completion of treatment, for stages I-IIA, patients were followed up every 6 months for five years and then annually as clinically indicated. For patients with stage IIB to IV, these were followed every three months for a total of two years, then every six months for three additional years, then annually depending on the clinical status of the patient. For patients with stage IIB to IV, every patient has scheduled computed tomography (CT) scans with intravenous contrast and ultrasounds of the adjacent lymph node regions every 12 months for three years, then additional CT scans or ultrasounds are performed if the patient presents symptoms. We have included these statements in our materials and methods.

**Reviewer’s comment:**

**2.**what was the patient compliance to follow up and attrition rate?

**Authors’ response:**

Thank you very much for this comment to improve our manuscript. The attrition rate was 5.1% at 5 years, and 17.5% at 10 years. The compliance to follow-up is challenging to measure in a retrospective study such as number of visits that they attended. Despite we do not have this information in our study, the fact that this was done at a national cancer center, patients are encouraged to adhere to follow-up appointments and were contacted in the event of a missed appointment. These statements have been included in our materials and methods and limitations.

**Reviewer’s comment:**

**3.**How the patients were managed( advanced cases) as many of them would have progressed in absence of immunotherapy.

**Authors’ response:**

Thank you for this comment. Due to unavailability of immunotherapy in our country, advanced cases were treated with a combination of chemotherapy, interferon, and radiotherapy, and surgical resections for locoregional recurrences. No patients received neoadjuvant therapies and the adjuvant chemotherapy regimen administered was dacarbazine. Therefore, we had worse survival outcomes (overall and event free survival) compared to other countries with immunotherapy available. Indeed, as our results showed, most patients with advanced disease experienced disease progression and worse survival rates than those diagnosed at early stages. We have included these statements in our discussion.

**Reviewer’s comment:**

**4.**Since AJCC ( 8th) edition has been used..how have the older patients (5 years back cases) classified??Have this change been taken care of while the data has been compiled?

**Authors’ response:**

We appreciate the comment of the reviewer. We agree that we collected data from patients that were diagnosed before the AJCC 8th edition was published. For this reason when medical records were reviewed, all the cases were re-classified according to the AJCC 8th edition. Each case was reviewed with clinical and pathological data to perform an accurate staging classification. We have included this in our materials and methods.
