# Supplementary material for: Melanoma in Peru: 1000 patients and 10 years of experience
Source: PLoS One. 2025 Oct 8;20(10):e0323636. doi: 10.1371/journal.pone.0323636 (PMC12507319; doi:10.1371/journal.pone.0323636)
Supplement: Attachment — Submitted filename: Response to reviewers 8-25-25.docx [file pone.0323636.s005.docx]

**EDITOR**

**Reviewer’s comment:**

Please respond to all reviewer comments and revise the manuscript accordingly. On my end, I kindly ask you to review the submitted table (Melanoma Data Final); some cells display the value '#NULL!'. Please clarify whether these represent missing data or if they are due to an error. Additionally, include a comprehensive data dictionary for all variables used.

**Authors’ response:**

We appreciate the comments provided. #NULL! Means that these values were missing data, we have kept them as NULL rather than leaving those values in blank. We have also mentioned in the methods section that we have excluded these missing values from the analysis. We have also included a comprehensive data dictionary for all variables used.

**Reviewer’s comment:**

I also understand the importance of having at least a five-year follow-up period (2019–2024), which may justify the end of the inclusion period; however, this should be clearly stated and discussed in the manuscript.

**Authors’ response:**

We appreciate this comment to improve our manuscript. We have added this statement to our materials and methods.

**Reviewer’s comment:**

Furthermore, the survival curves must include the associated p-values to allow assessment of whether there are statistically significant differences between groups.

**Authors’ response:**

We appreciate this valuable comment and have added the p-values to the survival curve figures.

**REVIEWER 1**

**Reviewer’s comment:**

Reviewer #1: Why was the data only collected up to 2019? It would be reasonable to expect inclusion of more recent data. It may be interesting to observe any changes that may have occurred following the COVID-19 pandemic.

**Authors’ response:**

We appreciate the comment of the reviewer, the main reason was to have at least five-year follow up period, being the last year included 2019. We agree with the reviewer’s observation about the changes during COVID-19 pandemic, we have included this in our limitation and proposed as a future study.

**Reviewer’s comment:**

Could you show the prevalence over the years?

**Authors’ response:**

We thank the reviewer for the suggestion. We have now included a figure (Figure 2) showing the yearly prevalence of cases from 2010 to 2019, which allows easier comparison of prevalence across the years. We have also included these findings in our results section, a figure (Figure 2) and have added a paragraph to discuss these findings.

**Reviewer’s comment:**

Also, the terms univariate/multivariate analyses would be more accurately described as univariable/multivariable analyses in this context.

**Authors’ response:**

We thank the reviewer for this valuable comment, we have corrected this statement throughout our manuscript.

**Reviewer’s comment:**

Although Kaplan-Meier analysis was mentioned, the results were not presented.

**Authors’ response:**

We appreciate the reviewer’s comment. These documents were originally uploaded as images; we have now included the images within the manuscript, separate from the uploaded documents, to improve understanding. These results were mentioned in page 12 before Table 4.

**REVIEWER 2**

**Reviewer’s comment:**

Reviewer #2: The authors present a very well organized and analyzed retrospective cohort study of melanoma in Peru, all of the issues raised by previous reviewers were adequately addressed.

**Authors’ response:**

We appreciate the comments of the reviewer.

**REVIEWER 3**

**REVIEWER 3**

Reviewer #3: (No Response)

**REVIEWER 4**

**Reviewer’s comment:**

Reviewer #4: This is a well done study that sheds light on the sociodemographic, clinicopathological features, and long-term outcomes such as event-free survival (EFS) and overall survival (OS) Peruvian population with melanoma.

However, there were some limitations of this study that should be discussed by the authors in the interpretation of their findings. The authors should indicate in the Materials and methods section how the specimens was confirmed by the Pathology department of the institute (Breslow thickness exclusively and/or IHC markers ?) for a more precise assessment of the tumoral thickness. The use of immunohistochemical markers for melanoma diagnosis rather than gene expression signatures data for classification of melanoma subtypes was an obvious limitation of this study that should be considered in the Discussion section.

**Authors’ response:**

We appreciate the reviewer’s valuable comments to improve our manuscript. Specimens were confirmed by the Pathology Department of our institute, with all cases reviewed by at least two pathologists. Tumor thickness was assessed according to Breslow depth. In cases where histopathological evaluation alone was insufficient, immunohistochemical (IHC) markers were employed to support the diagnosis of melanoma. Unfortunately, gene expression signatures are not available in public institutions in our country. These statements have been added to the Materials and Methods section, and the lack of molecular classification has been acknowledged as a limitation in the Discussion section.
